# The Treatment of Fractures from a Common-Sense Point of View

**Published:** 1907-08-31

**Authors:** 


### THE TREATMENT OF FRACTURES FROM A COMMON-SENSE POINT OF VIEW.

### III. OPERATIVE TREATMENT.

In last week's article the indications for mechanically fixing the fractured ends by wiring or by some similar manœuvre were briefly indicated. It may be added that such measures are undertaken either as a primary method of treatment or when the ordinary means of retaining the fragments in apposition have been tried but have failed. In the former case the operation should be undertaken as soon as the limb can be rendered aseptic. It is a matter of vital importance to obtain primary union after operations in which bone has been united by wire. The limb must be thoroughly cleaned with a scrubbing brush and ether soap, and then wrapped in gauze which has been soaked in a 1 in 2,000 solution of biniodide or perchloride of mercury. This dressing may be left on with advantage for 48 hours.

### The Dangers of Wiring.

The result of infection of these wounds is most disastrous. Periostitis is set up in the ends of the bone, and it is not uncommon to find that the portions of bone distal to the wire in each fragment separate from the shaft and come away as a sequestrum. An interval is thus left between the fragments, so that even if firm union is ultimately obtained, which is not always the case, there is bound to be an appreciable amount of shortening in the limb. It will therefore be readily understood that it is inadvisable to wire the fragments in compound fractures immediately after the accident, as is often done. It does not necessarily follow that because a fracture is compound the ends of the bone should be mechanically fixed. In the absence of one of the indications already mentioned, it is as easy to obtain a good result by using less radical methods. In compound fractures the wound is nearly always contaminated with organisms, either from the road on which the patient fell or from portions of his clothing; and if it is necessary to wire the fragments, it is better to wait for three days to make sure that the wound is aseptic before undertaking any such measures. In the case of a fracture of the patella, it is generally necessary to wire the fragments if a useful limb is to be obtained, since treatment by the older methods often leads to fibrous union and much loss of power in the limb; and here it is more than ever essential that strict asepsis should be observed. There is no more disastrous accident in surgery than septic infection of the knee-joint. The synovium, unlike the peritoneum, which has considerable bactericidal power, seems to have no resistance to pyogenic organisms; and the patient is likely to lose, in proportion to the severity of the infection, his power of movement at the joint or his limb, or even in bad cases, his life.

### The Operation.

The technique of the operation for wiring a fractured bone is comparatively simple. An incision is made in the axis of the limb over the site of the fracture, and this must be long enough to enable the surgeon to see exactly what he is doing. The muscles are then held apart and the ends of the bone exposed. Each end is drilled obliquely from without inwards by means of a bradawl or Archimedean drill. The bradawl is the simpler instrument, but the Archimedean drill saves much trouble and labour in the case of a compact bone like the tibia. The points to be observed in drilling the bone are (1) the periosteum at the end of the bone must not be damaged more than is absolutely necessary, lest a portion of bone deprived of its periosteal blood-supply should necrose; (2) the drill must be entered at some distance from the end of the fragment—roughly, from half an inch to an inch—or the bone is apt to split; (3) the holes must be drilled so that the points of exit of the drill shall be exactly opposite each other when the fragments are in apposition. A piece of silver wire is now introduced, from the periosteal to the medullary surface in one fragment and in the opposite direction in the other. While the fragments are held in apposition the wire is tightened up by twisting its extremities, and the twisted portion is hammered down flat along the surface of the bone. This method can be applied to nearly all fractures of long bones; but some surgeons advocate the use of silver screws to fasten the ends together in very oblique fractures, while others fix the ends in transverse fractures by means of aluminium plates which are nailed to the shaft of the bone above and below the site of fracture. The limb is put up in splints until union has taken place.

### After Treatment.

When a fractured limb has been fixed in a retentive apparatus it still demands constant attention if a good result is to be obtained. If it is merely left until the bone is united, it will be found that the muscles of the limb are much atrophied from disuse; and, especially if the site of fracture is in the neighbourhood of a joint, there may be limitation of the movements of that joint; and an appreciable length of time elapses before the patient recovers complete use of the limb. To obviate these disagreeable consequences massage and passive movements are invaluable. The tendency to-day is to make much earlier and much freer use of these manipulations than was formerly the case.

